# 3D LiDAR-Based Precision Vehicle Localization with Movable Region Constraints

**DOI:** 10.3390/s19040942

**Published:** 2019-02-22

**Authors:** Chih-Ming Hsu, Chung-Wei Shiu

**Affiliations:** Graduate Institute of Mechatronic Engineering, National Taipei University of Technology, Taipei 106, Taiwan; t103408054@ntut.edu.tw

**Keywords:** localization, normalized cross-correlation, 3D LiDAR

## Abstract

This paper discusses a high-performance similarity measurement method based on known map information named the cross mean absolute difference (CMAD) method. Applying the conventional normalized cross-correlation (NCC) feature registration method requires sufficient numbers of feature points, which must also exhibit near-normal distribution. However, Light Detection and Ranging (LiDAR) ranging point cloud data scanned and collected on-site are scarce and do not fulfill near-normal distribution. Consequently, considerable localization errors occur when NCC features are registered with map features. Thus, the CMAD method was proposed to effectively improve the NCC algorithm and localization accuracy. Because uncertainties in localization sensors cause deviations in the localization processes, drivable moving regions (DMRs) were established to restrict the range of location searches, filter out unreasonable trajectories, and improve localization speed and performance. An error comparison was conducted between the localization results of the window-based, DMR–CMAD, and DMR–NCC methods, as well as those of the simultaneous localization and mapping methods. The DMR–CMAD method did not differ considerably from the window-based method in its accuracy: the root mean square error in the indoor experiment was no higher than 10 cm, and that of the outdoor experiment was 10–30 cm. Additionally, the DMR–CMAD method was the least time-consuming of the three methods, and the DMR–NCC generated more localization errors and required more localization time than the other two methods. Finally, the DMR–CMAD algorithm was employed for the successful on-site instant localization of a car.

## 1. Introduction

Autopilot technology was developed to substantially improve driving safety and convenience and thereby mitigate the burden of drivers. In the future, fully automated vehicles will likely constitute the main body of the smart transportation system and replace human drivers entirely; however, in its current form, this technology is more moderately implemented in advanced driver assistance systems. One mandatory function of autopilot technology is environmental perception, which prevents collision. Similarly crucial is accurate localization, particularly in urban environments where vehicles are operated on city roads; like human drivers, automated vehicles must adhere to traffic rules.

The existing self-localization systems in vehicles are dependent on sensor assistance, and they are categorized into passive or active sensor systems according to the types of sensors used. Passive sensor systems are further divided into global navigation satellite systems, inertial navigation systems, and stereo visions. The accuracy of global navigation satellite systems is affected by non-line-of-sight reception and multipath interference [1]; when operating in indoor environments, they also may not receive consistent signals, hampering their ability to provide location information. Inertial navigation systems, which provide accurate relative location of vehicles instantly, are subject to deteriorating accuracy over time [2]. Stereo visions involve the use of vision-based lane detection and provide location information by detecting stop lines [3], curbs [4,5], arrows [6,7], and traffic signals [8]. However, in poorly lit environments, such as indoor parking lots, the localization accuracy of stereo visions may decrease; moreover, these systems cannot locate vehicles on the road without the aforementioned signs.

The existing active sensors comprise laser range finders and light detection and ranging (LiDAR) systems, the latter of which are more commonly used. Prior research on smart vehicles has predominately discussed the successful implementation of two-dimensional (2D) LiDAR and Velodyne active sensor systems. Overall, active sensors are widely favored over passive sensors because they simplify the distance estimation processes of basic distances and generate desirable localization results.

Simultaneous localization and mapping (SLAM) systems simultaneously illustrate and update maps of unknown environments and locate agents. SLAM is a primary component of robot navigation systems, and has considerably evolved over the past two decades. As its name implies, SLAM systems provide both localization and mapping functions. The localization functions comprise curb-based, road mark-based, and landmark-and-building-based localization. Hata et al. [9] developed a curb-based localization technique using Velodyne sensors, wherein curbs were identified as obstacles and detected through the multilayer laser ring compression in the LiDAR. Subsequently, Hata et al. [10] proposed a road mark-based localization technique, again using Velodyne sensors; in this method, all the road signs on road surfaces were captured using the intensity information in the LiDAR for localization. A landmark-and-building-based localization technique, also using Velodyne sensors, was then established by Choi [11]. He developed a mixed-map SLAM system to illustrate environments through the simultaneous employment of grid and feature maps, which consist of both 2D and three-dimensional (3D) mapping. The GraphSLAM algorithm is used for 2D mapping and was regarded as a least-square problem by Thrun et al. [12]. In large-scale mapping, GraphSLAM can process a massive number of features and integrate global positioning system information to its mapping processes. For example, Levinson [13] combined a global positioning system, an inertial measurement unit (IMU), an odometer, and LiDAR data to generate high-resolution 2D surface maps. However, 3D mapping is more reliable and accurate than 2D mapping. Particularly, featureless roads benefit the most when preprepared maps are used for navigation because they mitigate the cumulative errors in SLAM, whose instant localization results may be undesirable for these roads.

Previous studies have incorporated various estimation technologies to solve the problems in SLAM. Smith and Cheeseman [14,15] developed the extended Kalman filter (EKF) to solve these problems. However, when the road mark information in the EKF increases, its covariance matrix may expand and aggravate calculation load; in short, road mark localization errors can escalate into substantial cumulative errors in the EKF. Moreover, the EKF is only suitable for solving linear systems; when used to solve nonlinear systems, the EKF may lead to slow convergence or undesirable divergence. Subsequently, Montermerlo et al. [16,17] developed FastSLAM, which is based on particle filters. FastSLAM 1.0 employed only basic odometer information to estimate the location of a robot, and thus the estimation accuracy of the system decreased following an increase in the cumulative errors in the odometer. By contrast, FastSLAM 2.0 applied the concept of EKF updates and the linearization of nonlinear environmental information to improve its localization accuracy. However, using the EKF to update the location of the robot increased the quantity of the environmental information and the calculation cost. 

For a 3D point cloud map, existing approaches, including Normal Distribution Transform (NDT) [18,19,20,21], Iterative Closest Point (ICP) [22,23,24], and Monte Carlo localization (MCL) [25,26,27,28,29,30,31] can be adopted as the map matching module. NDT divides the point cloud space into several grids and calculates their normal distribution, after which it analyzes the optimal solution of the transfer by calculating the probability distribution function. NDT requires more point cloud data than other methods do because NDT analyzes the transfer relationship through their distribution, and the large amount of data results in a slow calculation speed. ICP is currently the most commonly used algorithm in scan matching; it searches for the nearest points between two point clouds and analyzes their transfer relationship through singular value decomposition. However, ICP is used to find locally optimal solutions, and both favorable point cloud data and initial values are required to yield a relatively satisfactory convergence. Three problems with the conventional Monte Carlo localization (MCL) algorithm [25,26] still need to be addressed. Specifically, when the number of particles is difficult to determine, or when the particles are assigned excessive weights, the algorithm may generate only local optimal solutions, and the robot cannot have its location restored after it is entrapped in such solutions. Improvements on MCL, such as the Kullback–Leibler divergence MCL [27,28] and self-adaptive MCL [30,31], have been created to mitigate these problems; however, the problems surrounding direction estimation remain. Furthermore, MCL involves the use of random particles and is inapplicable for instant localization. To solve the aforementioned problems, preprepared maps can be used for instant mapping. In this study, 3D LiDAR was employed for 3D environmental perception, a map database was preprepared, and an algorithm was designed for instant and accurate indoor and outdoor localization. Moreover, there exist a number of range sensor models in the literature that measure cross-correlations [32,33] between a measurement and the map. A common technique is known as map matching. Map matching techniques provide the ability to transform scans into maps. Once both maps are in the same reference frame, they can be compared using the map cross-correlation function. Applying the conventional normalized cross-correlation (NCC) feature registration method requires sufficient numbers of feature points, which must also exhibit near-normal distribution. However, LiDAR ranging point cloud data scanned and collected on-site are scarce and do not fulfill near-normal distribution. Consequently, considerable localization errors occur when NCC features are registered with map features.

Earlier, Chong et al. [34] proposed the synthetic LiDAR, in which synthetic 2D information was scanned according to 3D features and the problems in localization and mapping were solved through 2D methods. A 3D rolling window was used to reestablish the 3D environmental information and the surface normal vector was calculated. Subsequently, the 3D point cloud was divided into blocks. A threshold value was determined to preserve the feature points perpendicular to the ground and project them to a virtual 2D plane, thereby completing the construction of the synthetic LiDAR model.

The present study employed the synthetic LiDAR as its basic framework, proposed a high-robustness similarity measurement method measuring cross mean absolute difference (CMAD), and integrated the CMAD in the drivable moving regions (DMRs) for instant localization. This localization method detected moving objects more satisfactorily than did the conventional normalized cross-correlation (NCC) method. Notably, only the 3D LiDAR was employed herein, and no additional odometer or IMU data were incorporated for support. Therefore, the search method did not feature a motion model, and the vehicle travel information could not be accurately identified. Furthermore, when the window-based method was employed for localization, the estimated location would suddenly shift sideways or backwards; hence, DMRs were added to restrict unreasonable movement regions, filter inappropriate localization trajectories, and improve the speed and performance of the localization system. According to the results, although the DMR method did not markedly differ from the window-based method in its localization accuracy, it required less time to locate the vehicle than did the window-based method. Additionally, when a particle filter was used for localization, the particles were not required to be spread across the entire window region; rather, they were only required to be spread to the farthest possible drivable region, thereby shortening the convergence time and attaining instant localization. 

## 2. Method 

### 2.1. Procedural Structure of the Localization Algorithm

**Figure 1:**
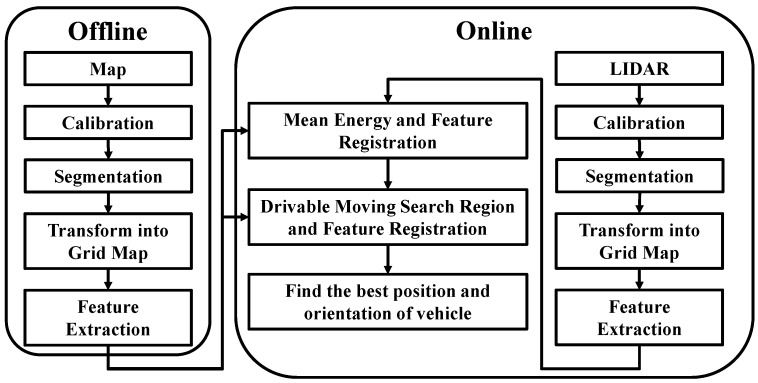
Flow chart of the localization algorithm.

Figure 1 illustrates the structure of the map localization algorithm. A 3D point cloud was mapped, calibrated, and segmented to obtain the required point cloud information, and the information was then transformed into a grid map. Finally, a virtual LiDAR scanning method was employed to extract environmental features and establish a map database.

The aforementioned procedures were also employed for the LiDAR on-site scanning process. After all the features were extracted, the initial location and direction were estimated through the mean energy method, and the DMR was incorporated in the feature registration process to identify the current location and direction of the vehicle. 

### 2.2. Calibration

**Figure 2:**
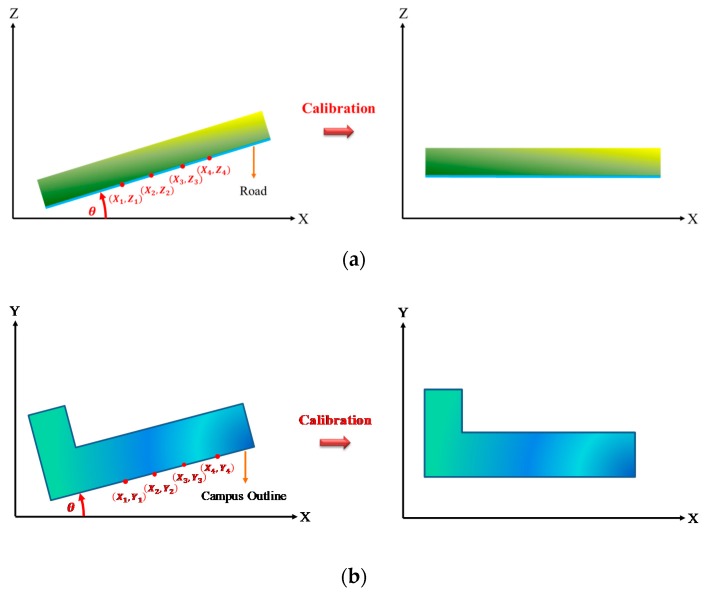
Calibration of the 3D point cloud. (**a**) Divergence in the Light Detection and Ranging (LiDAR) pitch angle. (**b**) Divergence between the LiDAR heading angle and the head direction of the vehicle.

In the experiment, the position of the LiDAR was not calibrated and thus diverged from the road surface, leading to the point cloud information pattern depicted in Figure 2. Therefore, four points were selected from the *X–Z* plane of the point cloud, portrayed in Figure 2a, and two individual points were selected for the slope calculation in (1). The inverse trigonometric function $\tan^{-1}$ was then employed to calculate the angles $\varphi_{A}$ and $\varphi_{B}$, as shown in (2). Subsequently, the mean between the two angles was obtained to identify the angle of rotation θ, as shown in (3), and the entire 3D point cloud was calibrated for easy map database construction. Figure 2b depicts the divergence between the LiDAR heading angle and the head direction of the vehicle.

(1) $$Slope_{A}=\frac{Y_{3}-Y_{1}}{X_{3}-X_{1}};\;Slope_{B}=\frac{Y_{4}-Y_{2}}{X_{4}-X_{2}}$$

(2) $$\varphi_{A}={tan}^{-1}\left(Slope_{A}\right);\;\varphi_{B}={tan}^{-1}\left(Slope_{B}\right)$$

(3) $$\theta =\frac{\left(\varphi_{A}+\varphi_{B}\right)}{2}$$

### 2.3. Segmentation

**Figure 3:**
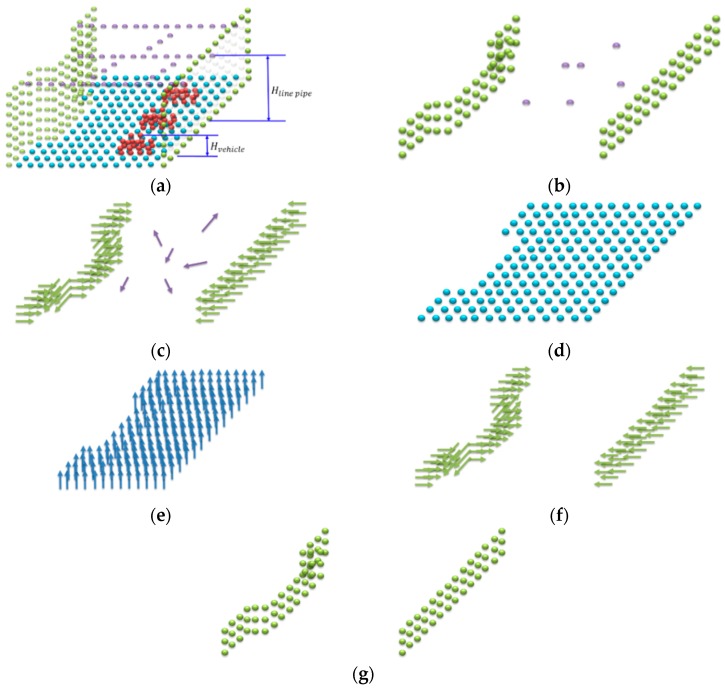
Segmentation of the 3D point cloud and its inner product. (**a**) Original point cloud information. (**b**) Point cloud information between $H_{vehicle}$ and $H_{line\;pipe}$. (**c**) Vector information of the segmented point cloud. (**d**) Point cloud information of the road. (**e**) Vector information of the road. (**f**) Vector information of the inner product. (**g**) Point cloud information of the inner product.

The calibrated point cloud was segmented to capture the features orthogonal to the road surface. Figure 3a illustrates the schematic of the original point cloud, in which the green, blue, red, and purple points represent walls, roads, vehicles, and pipelines, respectively. Only the environmental outline was needed in the experiment; the vehicle and pipelines were not required. Therefore, the cloud was segmented in a range larger than the height of the vehicle ($H_{vehicle}$) but smaller than that of the pipelines ($H_{line\;pipe}$), as outlined in Figure 3b. However, the desired outline may not be thoroughly presented after segmentation if miscellaneous points, such as the purple points depicted in Figure 3b, are still present.

The goal of segmentation was to obtain the inner product between the normal vectors of the segmented point cloud and those of the road point cloud. Therefore, the features perpendicular to the road must be retained. The inner product was within the range between the threshold values and represented the desired feature point. Calculating the inner product required segmenting the point cloud information of the road (Figure 3d), and calculating the normal vectors of the segmented point cloud and road point cloud. The vectors were calculated by fitting the least squares plane to the local neighboring points [35].

After the normal vectors of the segmented point cloud and road point cloud were calculated (Figure 3c,e) and the mean of the normal vectors of the road point cloud was obtained. Subsequently, the inner product between the normal vectors of the segmented cloud and the aforementioned mean was calculated. Finally, the threshold values were established for the inner product, and the point cloud information between −0.005 and 0.005 were retained, as indicated in Figure 3f,g.

### 2.4. Transforming the Point Cloud into a Grid Map

**Figure 4:**
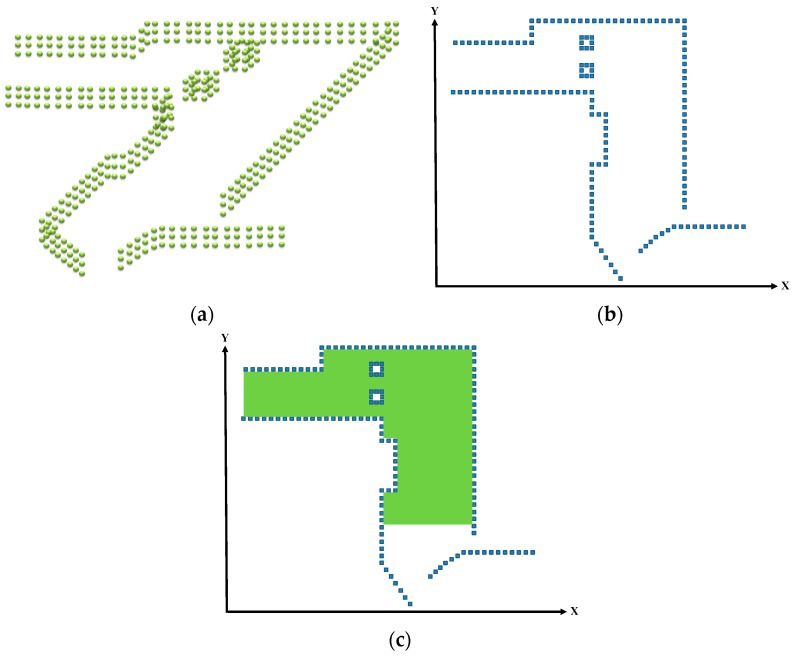
Transformation of the 3D point cloud into a 2D grid map. (**a**) Segmented point cloud. (**b**) Grid map. (**c**) Map database.

The 3D point cloud was transformed into a 2D grid map for extracting features and constructing a map database following its segmentation and calibration. Figure 4a presents the segmented 3D point cloud information, which was compressed into a grid map through transformation (Figure 4b). Environmental information about the walls and columns was contained in the grid map, where each grid was 10 × 10 cm^2^. Additionally, the grid map was incorporated to establish a map database as illustrated in Figure 4c, in which the green area represents the possible location of the vehicle; notably, this database featured *x*-axis, *y*-axis, and features information. The aforementioned procedures were also employed for the LiDAR on-site scanning process to capture the environmental features on-site, which were then registered with the feature data in the map database to identify the optimal location and direction. 

### 2.5. Feature Extraction

**Figure 5:**
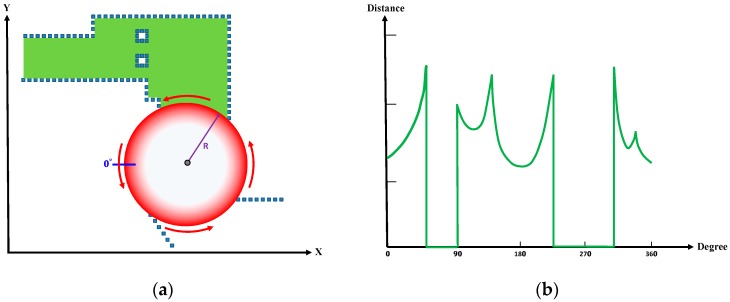
Extraction of the environmental features. (**a**) Virtual LiDAR scan. (**b**) Environmental features scanned within the radius R.

The environmental features were extracted from the grid map for their registration with the LiDAR data. Notably, virtual LiDAR scanning, rather than global scanning, was used to extract the features because locally scanned features are more accurate than those scanned globally. The features were scanned from within the circle with radius R (30% of the maximum scanning range), as depicted in Figure 5a; the initial scanning point (i.e., 0°) was on the left of the virtual LiDAR, and the data were scanned counterclockwise. The features extracted were expressed as distance–angle data (Figure 5b), in which the *x*-axis represents the angles and the *y*-axis represents the distances. Therefore, each cycle of LiDAR scan yielded 360 degrees of distance–angle data. Assuming that the grid database exhibited *N* possible vehicle locations, indicated by the green area in Figure 5a, each cycle of LiDAR scanning yielded 360 degrees of distance–angle data. Moreover, because each of the N locations exhibited different environmental features, the database featured a total of *N* × 360 feature data. These data were then incorporated into the map database containing only location information.

### 2.6. Feature Registration

The map and LiDAR features were registered to obtain the optimal location and heading angle of the vehicle in the follow-up localization calculation. The currently prevalent template matching technique involves conducting similarity measurement through the NCC method [36,37,38].

The reference and test signal were expressed as t and s, and its angle of movement on the test signal was displayed as τ. s(τ) represents a test signal with shifting τ angle. The NCC level of similarity was expressed as c(τ), which was calculated as shown in (4):

(4) $$c\left(\tau \right)=\frac{1}{N-1}\cdot \sum_{i=1}^{N}\frac{\left[t\left(i\right)-m_{i}\right]\left[s\left(i+\tau \right)-m_{s\left(\tau \right)}\right]}{\sigma_{t}\sigma_{s\left(\tau \right)}}$$

where $m_{i}$ and $m_{s\left(\tau \right)}$ represent the mean of t and s(τ), respectively; $\sigma_{t}$ and $\sigma_{s\left(\tau \right)}$ represent the standard deviation (SD) of t and s(τ), respectively; and *N* represents the degree of the reference signal (set as 360). This process was conducted through Fourier transform in the frequency domain to reduce calculation cost. Specifically, when c(τ) is maximized, τ represents the optimal matching angle between the test and the reference signal. The value of c(τ) ranges between −1 and 1: the closer it is to 1, the more similar the test and the reference signal are indicated to be.

**Figure 6:**
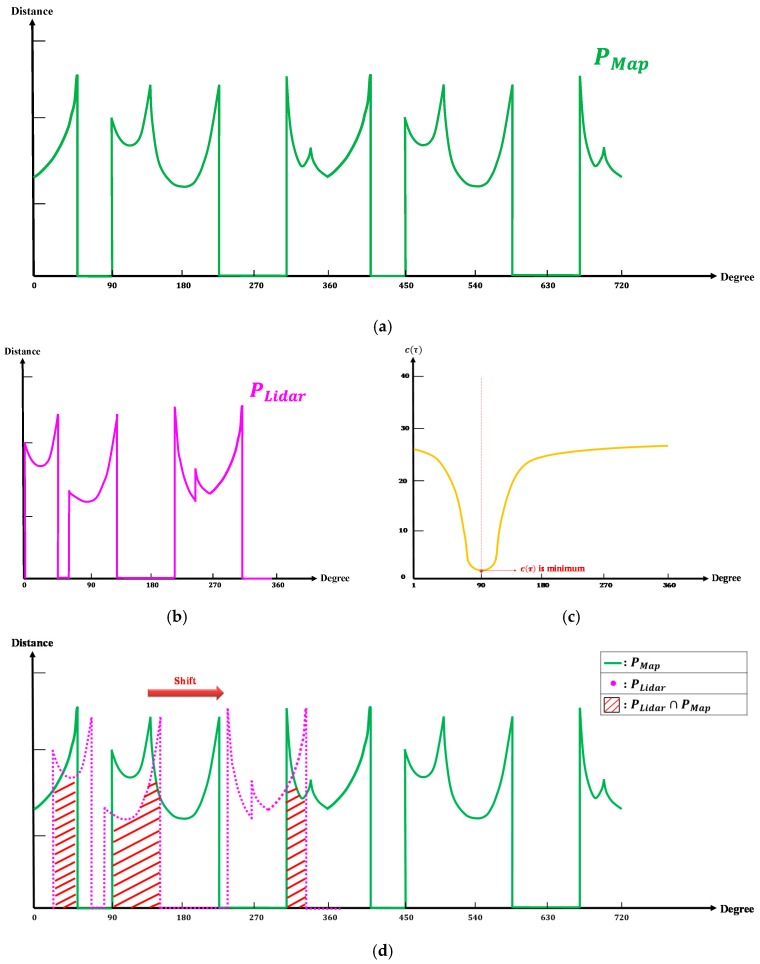
Registration of the map and LiDAR features. (**a**) Map features. (**b**) LiDAR on-site features. (**c**) Feature registration results. (**d**) Feature registration process.

Applying the NCC requires a sufficient number of matching points, which must also exhibit near-normal distribution. Herein, the point cloud information scanned through the LiDAR was relatively scarce. Consequently, a large localization error would result when the NCC features were matched with those of the LiDAR and the map. To enhance the NCC algorithm, the CMAD method was applied to compare the similarities between the map and LiDAR features. The parameters involved in this process are listed as follows

$$W=P_{Lidar}\cap P_{Map}\;P_{DMR}=\left\{p_{1},\;p_{2},\;p_{3},\;\cdots ,p_{L}\right\}\;V=\left\{P_{Best},\theta_{Heading}\right\}$$

where $P_{Lidar}$ represents the on-site LiDAR features (Figure 6b), $P_{Map}$ represents the virtually scanned LiDAR features (Figure 6a), W represents the set of the obstacle points scanned in both $P_{Lidar}$ and $P_{Map}$, $P_{DMR}$ represents the set of the location points in the DMR, and *V* represents the optimal location and heading angle selected through the poll mechanism.

As Figure 6d reveals, the template ($P_{Lidar}$) was overlapped with the fixed signal ($P_{Map}$) for feature registration. The angle of the template movement on the fixed signal is expressed as τ, and the similarity between the template and the signal is represented with c(τ), which was calculated as follows

(5) $$c\left(\tau \right)=\frac{1}{M}\sum_{i=1}^{M}\left|P_{Lidar}\left(i+\tau \right)-P_{Map}\left(i\right)\right|$$

where *M* represents the degree of *W* and τ ranges from $1^{{}^{\circ}}$ to $360^{{}^{\circ}}$. When c(τ) was minimal, the LiDAR and map features were the most closely matched (Figure 6c). Because (5) only involved the feature registration of one point, but the DMR featured a total of *L* points, the minimal c(τ) of the *L* points was used to determine the optimal location, $P_{Best}$, as shown in (6):

(6) $$P_{Best}=argmin\left\{P_{DMR}\left(c\left(\tau \right)\right)\right\}$$

where the corresponding τ represents the optimal heading angle $\theta_{Heading}$.

### 2.7. Estimating the Initial Location

The feature extraction was performed locally. Therefore, some angles did not show any distance value because no obstacles were scanned in that angle or that the distance values exceeded the scanning radius *R*. Next, both the LiDAR and map features were calculated through the mean energy method, which involves adding the distance values of all the scanned energy points together and dividing the sum by the number of energy points. Equation (7) measures the energy of the map, where *N* represents the number of energy points scanned, $d_{Map,i}$ represents the distance value of the *i*th degree, (8) measures the energy of the LiDAR, where M represents the number of energy points scanned, and $d_{Lidar,j}$ represents the distance value of the *j*th degree. Thus, the energy data of the map were established and implemented in the map database.

(7) $$E_{Map}=\frac{\sum_{i=1}^{N}d_{Map,i}}{N}$$

(8) $$E_{Lidar}=\frac{\sum_{j=1}^{M}d_{Lidar,j}}{M}$$

**Figure 7:**
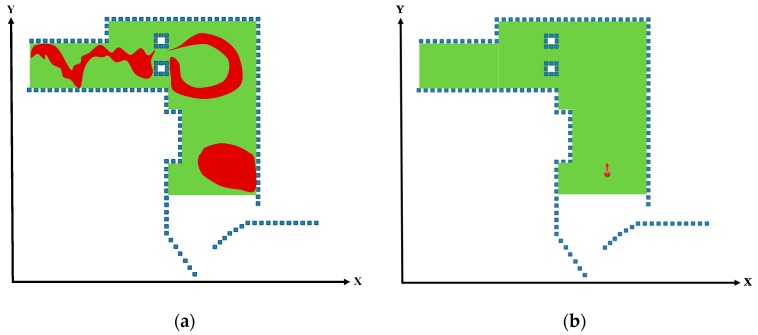
Identification of the initial location and direction of the vehicle through the mean energy method. (**a**) Possible vehicle locations identified through the mean energy method. (**b**) Initial location and direction identified through the feature registration.

The mean energy method was also applied to globally search the initial location and heading angle of the vehicle. The possible locations of the vehicle were selected through the aforementioned average energy values, where $E_{Lidar}$ is the energy of the LiDAR. A search range was set up to filter the energy of the map close to $E_{Lidar}$ as follows

(9) $$\left(E_{Lidar}-t\right)<E_{Map}<\left(E_{Lidar}+t\right)$$

where *t* represents the tolerable error, which was smaller indoors and larger outdoors. Because the point cloud of the map was more comprehensive than the on-site point cloud of the LiDAR (which was relatively scarce), most of the energy values of the map were higher than those of the LiDAR. Theoretically the initial location can be obtained by globally search the best registration of the map and LiDAR features. To reduce the computation time, the mean energy method was also applied to globally search the coarse initial location and heading angle of the vehicle. The possible locations of the vehicle were selected through the aforementioned average energy values. The selection results obtained using the mean energy method are indicated in red in Figure 7a. To acquire the optimal initial location and heading angle, the features from these selected positions were registered by CMAD with the LiDAR features that were scanned at the necessary instant (Figure 7b).

### 2.8. Window-Based Localization

**Figure 8:**
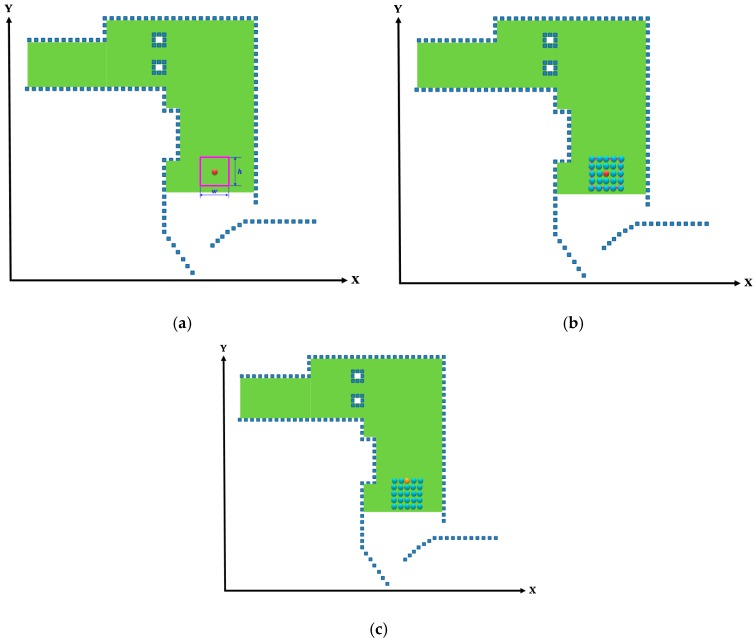
Locating the vehicle through window-based localization. (**a**) Window search area. (**b**) Current possible locations of the vehicle. (**c**) Final estimated location.

After the initial location of the vehicle was identified through the mean energy method, its location in the next time point was determined through window-based localization. Unlike the mean energy method, which was applied for a global search, window-based localization involved locally searching for the possible locations of vehicle through the window search method, and required considerably less calculation time. First, the initial location as calculated through the mean energy method was set as the center. A *w* × *h* window was created, depicted as the purple rectangular box in Figure 8a. Second, the possible locations of the vehicle at the current time point within the window were determined according to the coordinates of the initial location, and are displayed as the blue points in Figure 8b. Finally, the features of these locations were registered with the concurrent LiDAR features to obtain the current location of the vehicle, shown as the orange point in Figure 8c.

### 2.9. DMR-Based Localization

**Figure 9:**
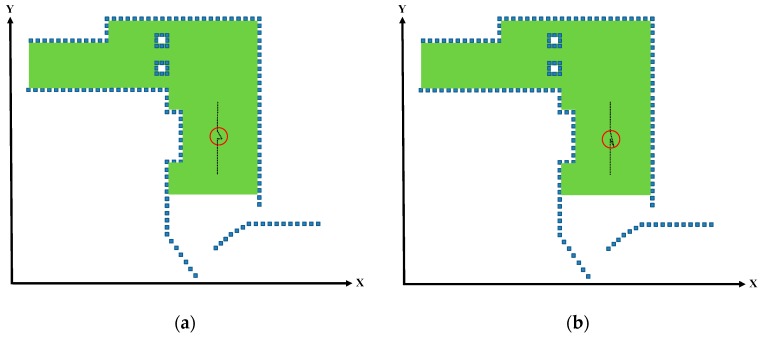
Deviations in window-based localization. (**a**) Sideways deviation in the window-based localization. (**b**) Backwards deviation in the window-based localization.

Because the location of the vehicle was searched using only 3D LiDAR without additional odometer or IMU data, and because the system did not include any motion model, the vehicle movement information could not be identified. Therefore, DMR-based localization was also conducted to calculate the trajectories that approximated to the actual vehicle movement. Although the motion trajectories that were estimated through the window-based localization could result in sideways or backwards deviations from the normal movement status, indicated by the red circles in Figure 9a,b, the DMR was designed to resolve this problem and reduce the number of estimated vehicle locations.

**Figure 10:**
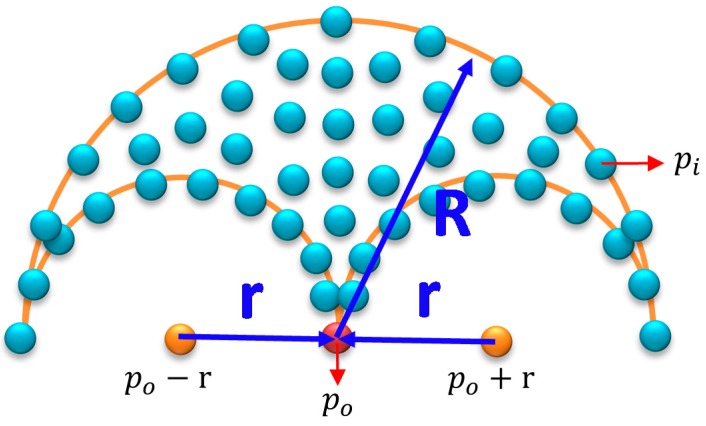
Drivable moving region (DMR).

**Figure 11:**
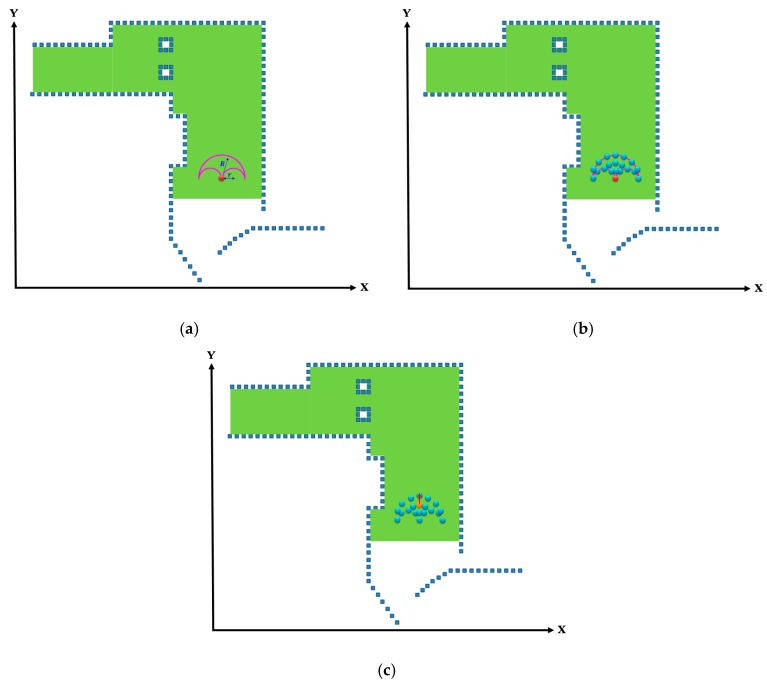
Locating the vehicle through DMR-based localization. (**a**) Search range of the DMR. (**b**) Possible current locations. (**c**) Estimated location and direction.

Similar to the window-based localization, the DMR-based localization involved estimating the initial location and direction of the vehicle through the mean energy method. Using this initial location as the center, the DMR with the maximal movement distance radius R and the rotation radius *r* was established (represented by the purple circle in Figure 11a). The entire DMR is illustrated in Figure 10, where $p_{o}$ represents the previously estimated location and the initial point. The distance between $p_{i}$ (a nearby location) and $p_{o}$ should be no longer than R, and the distances between $p_{i}$ and $\left(p_{o}+r\right)$ and between $p_{i}$ and $\left(p_{o}-r\right)$ must be longer than or equal to *r*. The $p_{i}$ that satisfied both these conditions was a point situated within the DMR, as determined by (10). Because the coordinate of the initial location was known, the other locations within the DMR could then be calculated; the possible current location of the vehicle is denoted by the blue points in Figure 11b. Unlike the window-based estimated locations, the DMR rotated according to the changes in the heading angle of the vehicle. Therefore, the heading angle from the previous time point was used as the angle of rotation, and the entire search area was rotated using the *z*-axis. Finally, the features of the locations in the area were registered with the concurrent LiDAR features to isolate the current location of the vehicle on the map, which is identified by the orange point in Figure 11c.

(10) $$\left\{\left|p_{i}-p_{o}\right|\leq R\right\}\cap \left\{\left|p_{i}-\left(p_{o}+r\right)\right|\geq r\right\}\cap \left\{\left|p_{i}-\left(p_{o}-r\right)\right|\geq r\right\}$$

## 3. Experiment

### 3.1. Equipment

**Figure 12:**
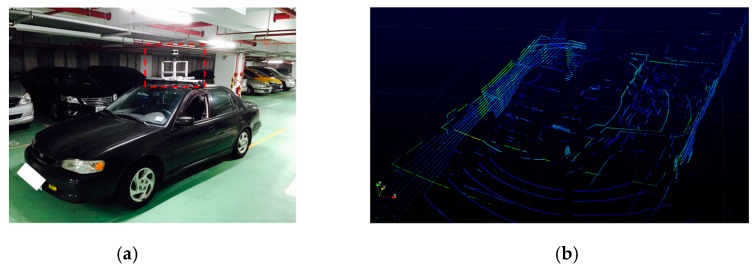
The aluminum extrusion support frame and a cloud deck of the LiDAR system. (**a**) Actual setup of the LiDAR. (**b**) On-site LiDAR point cloud.

Velodyne LiDAR (VLP-16) was employed to scan the surrounding environment using a laser, and establish a 3D point cloud. Notably, if the LiDAR sensor had been set up directly on the car, then it would have scanned the car itself because of the insufficient height. Therefore, the aluminum extrusion support frame attached with a cloud deck was heightened to thoroughly construct the 3D point cloud on the outlines and obstacles of the environment. Figure 12a depicts the actual installment of the sensor on the car, and Figure 12b illustrates the point cloud scanned on-site using the LiDAR.

### 3.2. Indoor Localization Experiment

**Figure 13:**
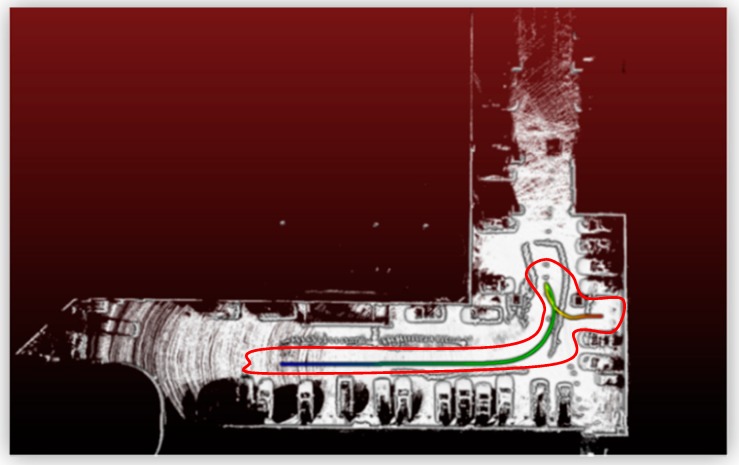
Indoor experiment environment.

The indoor environment selected for this study was the parking lot in Basement 5 of National Taipei University of Technology (NTUT). Figure 13 depicts the outline of the entire parking lot, and the motion trajectory of the car is circled in red. 

#### 3.2.1. Window-Based Localization

**Figure 14:**
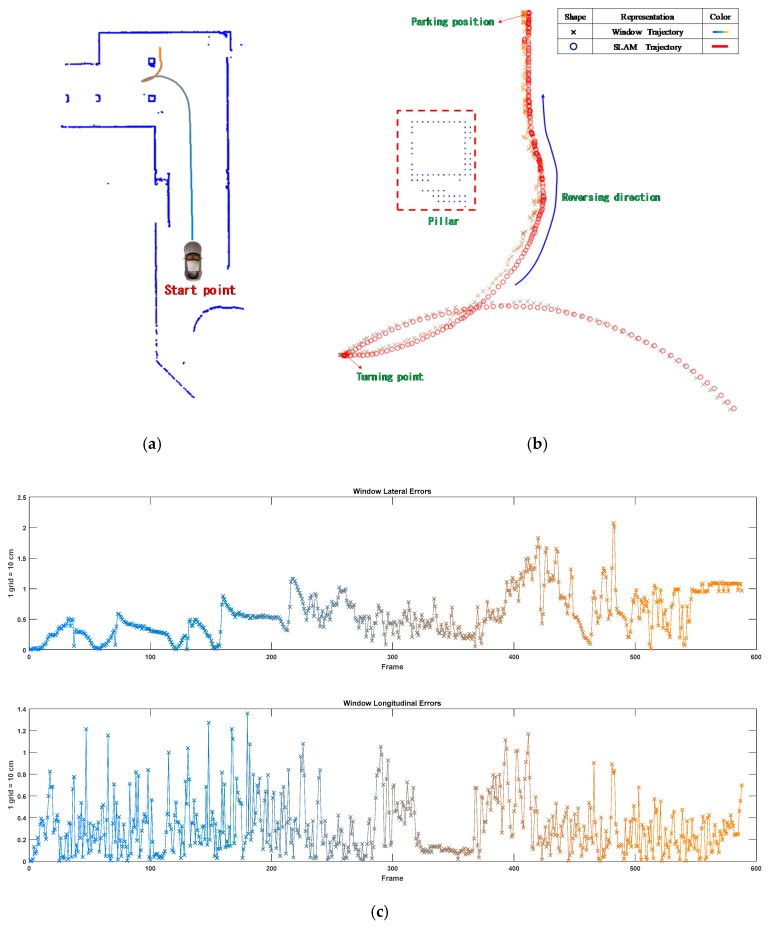
Comparison of the Window and SLAM trajectories. (**a**) Window trajectory. (**b**) Local magnification (when the car was reversing). (**c**) Lateral and longitudinal error comparison between the Window and simultaneous localization and mapping (SLAM) trajectories.

The motion trajectory detected through the window-based localization was smoothed using the EKF and compared with the trajectory identified through the SLAM method. Figure 14a depicts the trajectories identified through window-based localization. However, because the Window trajectory was considerably similar to the Kalman trajectory, the reversing sections of the trajectories were locally magnified (Figure 14b, where the SLAM trajectory is indicated by the circles and the Window trajectory is indicated by the crosses). Notably, when the car was reversing, sideways deviations occurred in the window-based localization. Figure 14c illustrates the lateral errors between the two calculated trajectories. Beginning at the 368th frame (i.e., when the car began reversing), the error substantially widened to a maximum of 2.07 grids (20.7 cm). Meanwhile, the maximal longitudinal error was approximately 1.36 grids (13.6 cm). 

#### 3.2.2. DMR-Based Localization

**Figure 15:**
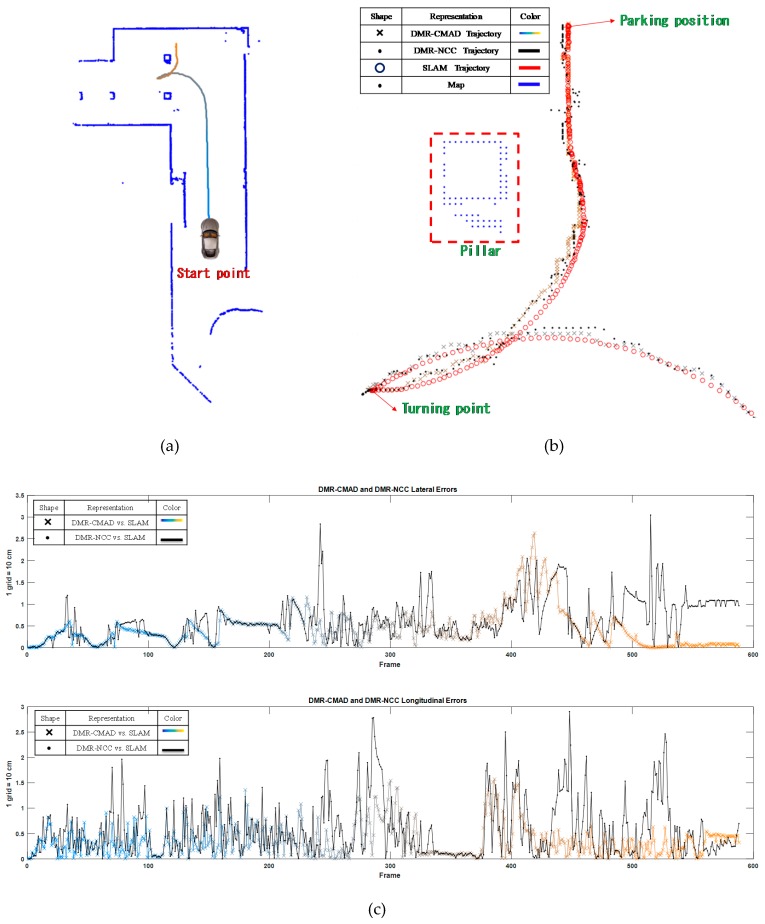
Comparison of the DMR–CMAD and DMR–NCC trajectories and the SLAM trajectory. (**a**) DMR–cross mean absolute difference (CMAD) trajectory. (**b**) Local magnification (when the car was reversing). (**c**) Lateral and longitudinal errors between the DMR–CMAD and DMR–normalized cross-correlation (NCC) trajectories and the SLAM trajectory.

The motion trajectory detected through the DMR-based localization was smoothed using the EKF and compared with the SLAM trajectory. The DMR–NCC trajectory was also compared with the SLAM trajectory. Figure 15a depicts the DMR–CMAD trajectory. However, because the three aforementioned trajectories were considerably similar, the reversing sections of the trajectories were locally magnified (Figure 15b, where the DMR–NCC trajectory is denoted by the dots, the SLAM trajectory is denoted by the circles, and the DMR–CMAD trajectory is denoted by the crosses). Notably, when the car was reversing, the DMR–CMAD trajectory was more stable than the Window trajectory, but the DMR–NCC trajectory exhibited considerable sideways errors. Figure 15c depicts the lateral errors between the three calculated trajectories. Beginning at the 372th frame (i.e., when the car began reversing), the errors substantially increased to a maximum of 2.63 grids (26.3 cm). The maximal longitudinal error was approximately 1.58 grids (15.8 cm). According to the error comparison between the DMR–NCC trajectory and the SLAM trajectory, changes in the errors were considerable during both periods when the car moved forward and when it reversed. The maximal lateral and longitudinal errors were approximately 3.05 grids (30.5 cm) and 2.9 grids (29 cm), respectively. The performance of the proposed DMR–CMAD algorithm is verified via vehicle tests on a parking-lot proving ground. The proposed algorithm will be useful in the implementation of autonomous self-parking control.

### 3.3. Outdoor Localization Experiment

**Figure 16:**
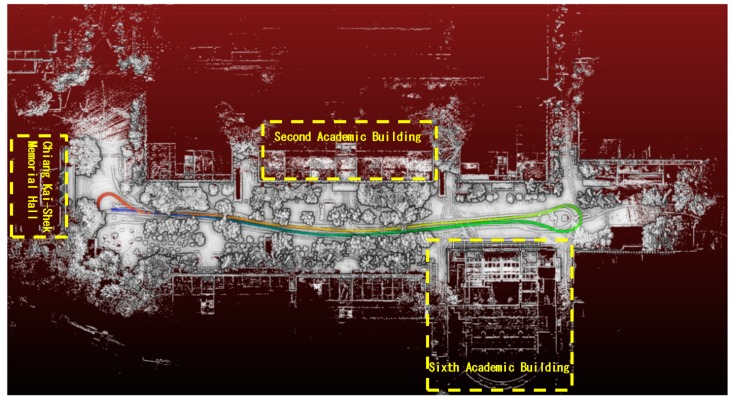
Outdoor experiment environment. 4. Experimental Results.

The outdoor environment for this study was the NTUT campus. Figure 16 presents a 2D outline of the campus, including the motion trajectory of the car. The car started from the front of Chiang Kai-Shek Memorial Building, made a U-turn around the spherical landmark in front of the Sixth Academic Building, traveled past the Second Academic Building through the original route, and stopped back in front of Chiang Kai-Shek Memorial Building.

#### 3.3.1. Window-Based Localization

**Figure 17:**
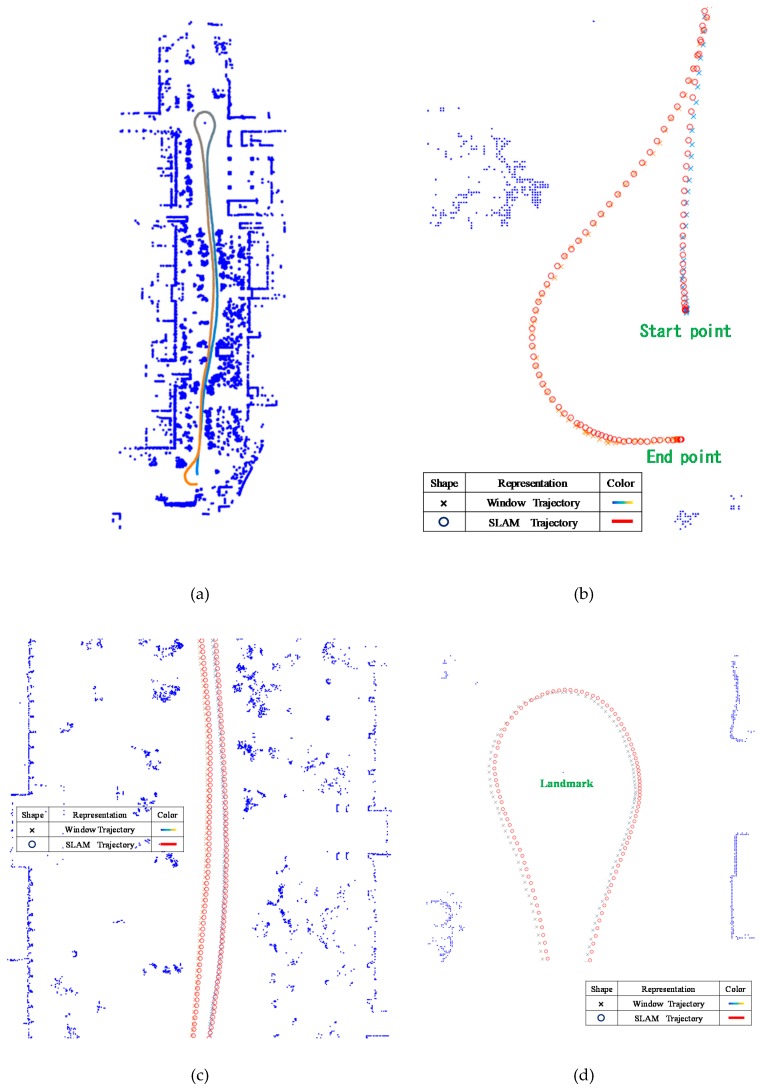

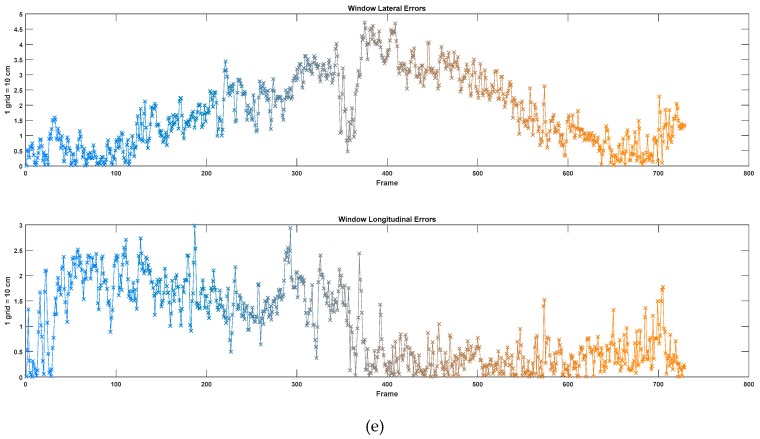
Comparison of the Window and SLAM trajectories. (**a**) Window trajectory. (**b**) Local magnification of the trajectory (start and end section). (**c**) Local magnification (middle section). (**d**) Local magnification (U-turn around the landmark). (**e**) Lateral and longitudinal errors between the Window and SLAM trajectories.

The motion trajectory detected through the window-based localization was smoothed using the EKF and compared with the trajectory identified through the SLAM method. Figure 17a depicts the Window trajectory. However, because the Window trajectory was very similar to the Kalman trajectory, three sections of the trajectories were locally magnified: Figure 17b displays the start and end sections; Figure 17c depicts the middle section; and Figure 17d illustrates the U-turn around the landmark. Specifically, the SLAM trajectory is denoted by circles and the Window trajectory is denoted by crosses. Notably, when the car was reversing, sideways deviations occurred in the window-based localization. As indicated by Figure 17e, the lateral errors from the 368th frame to the 409th frame (i.e., when the car made the U-turn) were the most substantial and the maximal lateral error was approximately 4.71 grids (47.1 cm). By contrast, the longitudinal errors were notable before the U-turn and decreased after the U-turn; the maximal longitudinal error was 2.99 grids (29.9 cm).

#### 3.3.2. DMR-Based Localization

**Figure 18:**
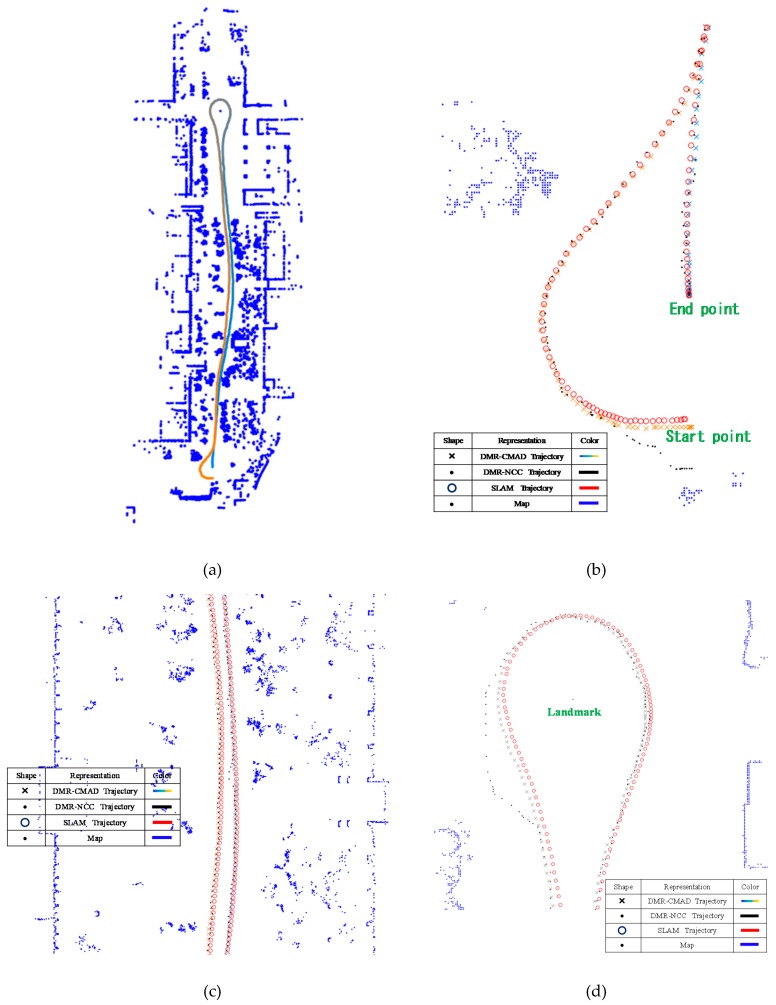

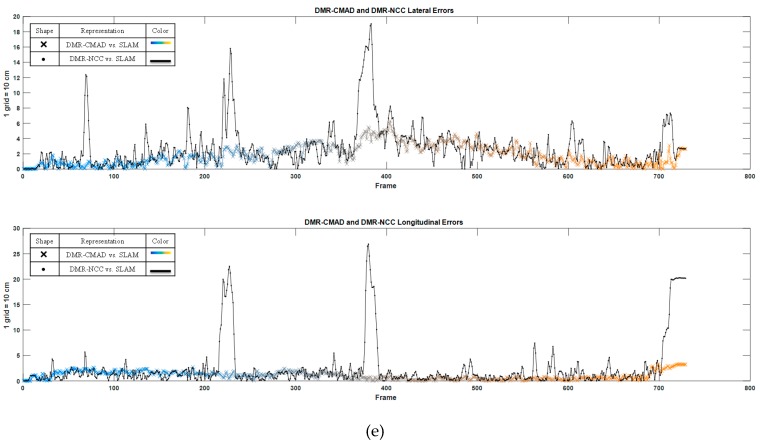
Comparison of the DMR–CMAD and DMR–NCC trajectories and the SLAM trajectory. (**a**) DMR–CMAD trajectory. (**b**) Local magnification (start and end section). (**c**) Local magnification (middle section). (**d**) Local magnification (U-turn around the landmark). (**e**) Lateral and longitudinal errors between the DMR–CMAD and DMR–NCC trajectories and the SLAM trajectory.

The motion trajectory detected through the DMR-based localization was smoothed using the EKF and compared with the trajectory identified through the SLAM method. The DMR–NCC trajectory was also compared with the SLAM trajectory. Figure 18a depicts the DMR–CMAD trajectory. However, because all three trajectories were very similar, three sections of the trajectories were locally magnified: Figure 18b displays the start and end sections, Figure 18c depicts the middle section, and Figure 18d illustrates the U-turn around the landmark. Specifically, the DMR–NCC trajectory is denoted by dots, the SLAM trajectory is denoted by circles, and the DMR–CMAD trajectory is denoted by crosses. Notably, when the car was reversing, sideways deviations occurred in the window-based localization. As indicated by Figure 18e, the lateral error at the 404th frame (i.e., when the car was making the U-turn) was the most substantial and the maximal lateral error was approximately 6.31 grids (63.1 cm). By contrast, the longitudinal errors were notable before the U-turn and decreased after the U-turn; the maximal longitudinal error, which was near the end point, was 3.34 grids (33.4 cm). Meanwhile, the variance in the localization errors of the DMR–NCC trajectory was substantial before the car turned, when the car turned, and when the car was approaching the end point. The maximal lateral error was approximately 19.04 grids (190.4 cm); the maximal longitudinal error was 26.9 grids (269 cm). The performance of the proposed DMR–CMAD algorithm is verified via vehicle tests on an outdoor proving ground. The proposed algorithm will be useful in the implementation of lane-level automated driving control.

### 3.4. Comparison of the Indoor and Outdoor Errors

The comparison of the root mean square errors (RMSEs) and SDs of the indoor and outdoor errors are outlined in Table 1. The accuracy of the DMR–CMAD trajectories approximated that of the Window trajectories, but the errors of the DMR–NCC trajectories, particularly those of the outdoor trajectory, were more substantial. 

In the experiment, Velodyne LiDAR (VLP-16) was employed to scan the surrounding environment using a laser and establish a 3D point cloud. The computer platform that was used for vehicle localization was equipped with an Intel i5 CPU, 8 GB DDRIII. Table 2 presents a list of the average localization time of all four methods examined in the indoor (totally 734 frames) and outdoor (totally 1274 frames) experiments. The localization time of the DMR–CMAD method was the shortest, the localization time of the DMR–NCC method was two to three times that of the Window and DMR–CMAD methods, and the localization time of the SLAM method was the longest. Notably, the localization times also varied between the indoor and outdoor environments, in part because the area of the indoor site was smaller and the speed the car was slower compared with the outdoor site.

## 4. Conclusions

This study employed the map-based DMR localization method to improve upon the sudden sideways and backwards deviations in the window localization algorithm. The DMR–CMAD and window methods did not differ substantially in their localization errors according to the RMSE and SD comparison results. However, the DMR–NCC method exhibited more errors and required more localization time than did the DMR–CMAD and window methods; the DMR–CMAD method was the least time-consuming of the four employed methods. Because the features of the outdoor experiment environment were more complicated than those of the indoor environment, the feature registration accuracy was slightly lower in the outdoor experiment than in the indoor environment, and the outdoor localization errors were markedly larger than the indoor localization errors. However, the localization accuracy of the DMR–CMAD method was overall ideal, and the method was confirmed as applicable for instant localization.

This study also incorporated the LiDAR to capture the environmental features on-site, which were then registered with the feature data in the map database to identify the optimal location and direction. However, if the features of future experimental sites differ from those of the map database, inaccurate localization may result. Furthermore, because Velodyne VLP-16 LiDAR was used to extract the on-site environmental characteristics, its number of point clouds was less than those of other 3D LiDAR systems. The point cloud densities in outdoor environments were relatively sparse, and the great variation of the point clouds between each frame resulted in substantial matching iteration error. Therefore, other types of sensors—GPS and IMU—can be used to reduce the problem of matching iteration error.

## Figures and Tables

**Table 1:** Lateral and longitudinal root mean square errors (RMSEs) and standard deviations (SDs) of the Window, DMR–CMAD, and DMR–NCC trajectories.

**Localization**	**NTUT B5 Parking Lot**		
**Method** **(1 grid = 10 cm)**	**Window**	**DMR-CMAD**	**DMR–NCC**
Lateral RMSE	0.67 grid	0.61 grid	0.79 grid
Standard Deviation	0.36 grid	0.42 grid	0.47 grid
Longitudinal RMSE	0.41 grid	0.48 grid	0.78 grid
Standard Deviation	0.26 grid	0.32 grid	0.55 grid
**Localization**	**NTUT Campus**		
**Method** **(1 grid = 10 cm)**	**Window**	**DMR-CMAD**	**DMR–NCC**
Lateral RMSE	2.16 grid	2.27 grid	3.91 grid
Standard Deviation	1.17 grid	1.33 grid	2.83 grid
Longitudinal RMSE	1.25 grid	1.31 grid	5.25 grid
Standard Deviation	0.75 grid	0.78 grid	4.63 grid

**Table 2:** Average localization times of the Window, DMR–CMAD, DMR–NCC, and SLAM methods.

**Location**	**NTUT B5 Parking Lot (734 Frames)**			
**Method**	**Window**	**DMR–CMAD**	**DMR–NCC**	**SLAM**
Time/Frame (s)	0.23	**0.2**	0.76	4.26
**Location**	**NTUT Campus (1274 frames)**			
**Method**	**Window**	**DMR–CMAD**	**DMR–NCC**	**SLAM**
Time/Frame (s)	0.7	**0.48**	1.03	3.45
